# Health Equity in Patients Receiving Durvalumab for Unresectable Stage III Non-Small Cell Lung Cancer in the US Veterans Health Administration

**DOI:** 10.1093/oncolo/oyad172

**Published:** 2023-06-19

**Authors:** Amanda M Moore, Zohra Nooruddin, Kelly R Reveles, Jim M Koeller, Jennifer M Whitehead, Kathleen Franklin, Paromita Datta, Munaf Alkadimi, Lance Brannman, Ion Cotarla, Andrew J Frankart, Tiernan Mulrooney, Xavier Jones, Christopher R Frei

**Affiliations:** Division of Pharmacotherapy, College of Pharmacy, The University of Texas at Austin, San Antonio, TX, USA; Pharmacotherapy Education and Research Center, Department of Medicine, Long School of Medicine, University of Texas Health San Antonio, San Antonio, TX, USA; Pharmacotherapy Education and Research Center, Department of Medicine, Long School of Medicine, University of Texas Health San Antonio, San Antonio, TX, USA; Division of Pharmacotherapy, College of Pharmacy, The University of Texas at Austin, San Antonio, TX, USA; Pharmacotherapy Education and Research Center, Department of Medicine, Long School of Medicine, University of Texas Health San Antonio, San Antonio, TX, USA; Research Service, Audie L. Murphy Memorial Veterans Hospital Division, South Texas Veterans Health Care System, San Antonio, TX, USA; Division of Pharmacotherapy, College of Pharmacy, The University of Texas at Austin, San Antonio, TX, USA; Pharmacotherapy Education and Research Center, Department of Medicine, Long School of Medicine, University of Texas Health San Antonio, San Antonio, TX, USA; Pharmacotherapy Education and Research Center, Department of Medicine, Long School of Medicine, University of Texas Health San Antonio, San Antonio, TX, USA; Research Service, Audie L. Murphy Memorial Veterans Hospital Division, South Texas Veterans Health Care System, San Antonio, TX, USA; Research Service, Audie L. Murphy Memorial Veterans Hospital Division, South Texas Veterans Health Care System, San Antonio, TX, USA; Pharmacotherapy Education and Research Center, Department of Medicine, Long School of Medicine, University of Texas Health San Antonio, San Antonio, TX, USA; Research Service, Audie L. Murphy Memorial Veterans Hospital Division, South Texas Veterans Health Care System, San Antonio, TX, USA; Pharmacotherapy Education and Research Center, Department of Medicine, Long School of Medicine, University of Texas Health San Antonio, San Antonio, TX, USA; Research Service, Audie L. Murphy Memorial Veterans Hospital Division, South Texas Veterans Health Care System, San Antonio, TX, USA; Oncology Business Unit, Global Medical Affairs, AstraZeneca Pharmaceuticals, Gaithersburg, MD, USA; Oncology Business Unit, US Medical Affairs, AstraZeneca Pharmaceuticals, Gaithersburg, MD, USA; Department of Radiation Oncology, University of Cincinnati, Cincinnati, OH, USA; Oncology Business Unit, US Medical Affairs, AstraZeneca Pharmaceuticals, Gaithersburg, MD, USA; Division of Pharmacotherapy, College of Pharmacy, The University of Texas at Austin, San Antonio, TX, USA; Research Service, Audie L. Murphy Memorial Veterans Hospital Division, South Texas Veterans Health Care System, San Antonio, TX, USA; Division of Pharmacotherapy, College of Pharmacy, The University of Texas at Austin, San Antonio, TX, USA; Pharmacotherapy Education and Research Center, Department of Medicine, Long School of Medicine, University of Texas Health San Antonio, San Antonio, TX, USA; Research Service, Audie L. Murphy Memorial Veterans Hospital Division, South Texas Veterans Health Care System, San Antonio, TX, USA

**Keywords:** lung cancer, durvalumab, health equity, health disparity, immunotherapy

## Abstract

**Background:**

Real-world evidence is limited regarding the relationship between race and use of durvalumab, an immunotherapy approved for use in adults with unresectable stage III non-small cell lung cancer (NSCLC) post-chemoradiotherapy (CRT). This study aimed to evaluate if durvalumab treatment patterns differed by race in patients with unresectable stage III NSCLC in a Veterans Health Administration (VHA) population.

**Materials and Methods:**

This was a retrospective analysis of White and Black adults with unresectable stage III NSCLC treated with durvalumab presenting to any VHA facility in the US from January 1, 2017, to June 30, 2020. Data captured included baseline characteristics and durvalumab treatment patterns, including treatment initiation delay (TID), interruption (TI), and discontinuation (TD); defined as CRT completion to durvalumab initiation greater than 42 days, greater than 28 days between durvalumab infusions, and more than 28 days from the last durvalumab dose with no new durvalumab restarts, respectively. The number of doses, duration of therapy, and adverse events were also collected.

**Results:**

A total of 924 patients were included in this study (White = 726; Black = 198). Race was not a significant factor in a multivariate logistic regression model for TID (OR, 1.39; 95% CI, 0.81-2.37), TI (OR, 1.58; 95% CI, 0.90-2.76), or TD (OR, 0.84; 95% CI, 0.50-1.38). There were also no significant differences in median (interquartile range [IQR]) number of doses (White: 15 [7-24], Black: 18 [7-25]; *P* = .25) or median (IQR) duration of therapy (White: 8.7 months [2.9-11.8], Black: 9.8 months [3.6-12.0]; *P* = .08), although Black patients were less likely to experience an immune-related adverse event (28% vs. 36%, *P* = .03) and less likely to experience pneumonitis (7% vs. 14%, *P* < .01).

**Conclusion:**

Race was not found to be linked with TID, TI, or TD in this real-world study of patients with unresectable stage III NSCLC treated with durvalumab at the VHA.

**Implications for Practice**

Real-world data are necessary to assess (1) subpopulations underrepresented in clinical trials and (2) the typical clinical use of new therapeutics outside of the highly controlled environment of a randomized controlled trial. Black patients shoulder a disproportionate share of the lung cancer burden in the US but are underrepresented in landmark immunotherapy trials. This analysis is the first study to evaluate the relationship between race and the clinical use of durvalumab and shows that in the Veterans Health Administration, an equal-access health care system, race is not linked with durvalumab treatment patterns of treatment initiation delay, interruption, or discontinuation.

## Introduction

The recent introduction of immune checkpoint inhibitors (ICIs) has led to a paradigm shift in the modern treatment landscape of lung cancer. In particular, the PACIFIC trial was a landmark clinical trial whose results led to the approval of durvalumab, an anti-programmed death-ligand 1 (PD-L1) monoclonal antibody, for use in patients with unresectable stage III non-small cell lung cancer (NSCLC) without disease progression after definitive chemoradiotherapy (CRT).^1^ However, clinical trials, while internally valid, are carried out under controlled conditions with homogenous patient populations that are typically not representative of real-world patients. Historically, White male participants have been overrepresented in randomized controlled trials, while women and racial and ethnic minorities have been underrepresented.^2-4^ Black patients shoulder a disproportionate share of the lung cancer burden in the US, yet only 2% of the intent-to-treat population in the PACIFIC trial was Black, thus limiting the generalizability of these findings.^1,5^

Lung cancer is the second leading cause of new cancer cases and the leading cause of cancer death in Black men and women, with Black men having the highest lung cancer death rate of any racial or ethnic group.^5,6^ Research efforts over the past decade have aimed at identifying the causes of these disparate survival outcomes and largely point to access-to-care as a major contributor to health disparities.^7-12^ Socioeconomic status (SES) is strongly correlated with race in the US and is a critical factor driving racial inequalities in cancer outcomes, as it impacts the ability to access high-quality health care and the receipt of optimal disease treatment.^6,9,13,14^ An increasing number of studies suggest that after adjusting for treatment, or in equal-access healthcare systems such as the Veterans Health Administration (VHA), race alone is not a predictor of outcomes, suggesting that efforts to equalize access-to-care and treatment might result in improved outcomes for Black patients with NSCLC.^12,15-20^

Given that Black patients have historically represented a small percentage of the population included in immunotherapy clinical trials for NSCLC but represent the group of highest burden in terms of incidence and mortality, real-world utilization studies may be helpful in identifying barriers to equitable care to ensure equal treatment. Currently, there is limited literature regarding health disparities in the use of ICIs for patients with NSCLC. Of the available studies, most are of small sample size from a single institution.^4,21,22^ Notably, there is an absence of studies examining disparities in durvalumab treatment. The purpose of this study was to evaluate racial disparities in durvalumab treatment patterns, including treatment initiation delay (TID), treatment interruption (TI), and treatment discontinuation (TD), and the associated reasons for such, between Black and White patients with unresectable stage III NSCLC at the VHA.

## Materials and Methods

### Study Design and Population

This was a retrospective cohort study of White and Black adult patients (≥18 years of age) with unresectable stage III NSCLC presenting to any VHA facility in the US from January 1, 2017, to June 30, 2020. This was an observational study that required no intervention or interference with standard medical care. This study was approved by the University of Texas Health San Antonio Institutional Review Board and the South Texas Veterans Health Care System Research & Development Committee. Patients for this study were first identified using structured electronic data. Inclusion criteria were (1) age 18 years or older, (2) inpatient or outpatient lung cancer diagnosis (ICD-10 codes C34X or D022X) between January 1, 2017, and June 30, 2020, and (3) an order for durvalumab (drug name, HCPCS C9492 or J9173, or NDC 0310-4500-12 or 0310-4611-50) between January 1, 2017, and June 30, 2020. Then, trained data abstractors used manual chart review to identify a subset of patients who satisfied the following inclusion criteria: (1) diagnosis of stage III NSCLC via pathology report during cohort inclusion period, (2) confirmation of unresectable tumor status, (3) receipt of CRT, and (4) initiation of durvalumab during inclusion period. Finally, trained data abstractors used manual chart review to exclude patients who met the following criteria: (1) non-NSCLC histology, (2) non-stage III tumor classification, (3) resectable tumor status, (4) durvalumab receipt preceding the study inclusion period, (5) durvalumab not received during the study period, (6) durvalumab therapy ongoing at the end of the study period, or (7) documented race other than White or Black.

Historical data were examined for one year prior to the study period to assess baseline characteristics and comorbidities. The index date was defined as the date of initiation of durvalumab. Patients were followed until the last VHA visit, loss to follow up, record of death, or the end of follow-up period on April 1, 2021, whichever occurred earlier (Supplementary Fig. S1).

### Study Variables and End Points

Structured electronic data were used for baseline variables including patient age, sex assigned a birth, race, Charlson score, comorbidities, VA priority group, distance from site of cancer care, and smoking status. Manual chart review was used to capture all other variables. All reasons for TID, TI, and TD, available in the notes, were captured, and a given patient could have more than one reason. Reasons were then sorted into predefined categories, such as “system issues” for those with reasons having to do with scheduling and coordination of care, “social reasons” for those with reasons including missed appointments and personal travel, and “other” for those with reasons identified outside of the predefined categories, including weather events and illnesses unrelated to their cancer diagnosis.

Race was obtained during patient enrollment and visit encounters; if different races were reported at separate encounters, patients were coded as the race that was most often reported. SES was captured as Veterans Affairs (VA) priority groups, a marker of SES based on service-connected disability, special status, and income level. Priority group 1 is representative of the most disability, with groups 2-6 representative of low-income patients, and groups 7-8 representative of the highest income levels.^23^ Geographic disparities in healthcare access were calculated using the mileage difference between patients’ residential zip code and the zip code of the nearest VHA site.

Patients were categorized as experiencing durvalumab TID if the time from CRT completion to durvalumab initiation was greater than 42 days (the maximum initiation time defined in the PACIFIC study).^1^ TI was defined as greater than 28 days between durvalumab infusions. Patients were reported to experience TD if more than 28 days passed from the last durvalumab dose with no new durvalumab restarts. A corrected duration of therapy (DOT) was calculated as DOT minus the days contributed by TI. The proportions of durvalumab-treated patients with a TID, TI, and TD were calculated as the number of patients experiencing each outcome divided by the total number of patients in each group. Reasons for a TID, TI, and TD and their proportions over the entire group were reported. Specific adverse events were pre-specified prior to data collection and were categorized as being immune-related or non-immune-related. The occurrence of an adverse event was only counted once per patient, even if multiple mentions of the event were found during chart review. Adverse events were only captured during durvalumab therapy, with no adverse events collected after durvalumab discontinuation or completion of planned treatment.

### Statistical Analysis

Analyses were conducted using JMP Pro 15 (SAS Institute, Cary, NC) with an alpha level set at *P* < .05. Bivariable statistical comparisons were conducted for White and Black patients using chi-square/Fisher’s exact tests and Student’s *t*-tests/Wilcoxon rank-sum tests. Multivariable logistic regression models were used to assess race as an independent risk factor for the study outcomes TID, TI, and TD. Divergent baseline characteristics (*P* < .1) and other relevant clinical and socioeconomic characteristics were incorporated as covariates in the model, with some categorical variables collapsed or substituted to maintain stability of the model while achieving parsimony. The following variables were included as covariates in all models: age, age-adjusted Charlson score, VA priority group, histology, Eastern Cooperative Oncology Group (ECOG) score, geographical distance from the nearest cancer care site, and smoking status. In the multivariable assessment of TID, time to first post-CRT imaging was also included as a clinically meaningful covariate. The results of the multivariable analyses were presented as odds ratios (ORs) and 95% confidence intervals (CIs).

## Results

A total of 1185 patients met the EHR inclusion criteria and 261 patients were excluded during chart review due to: non-NSCLC histology (*n* = 46), non-stage III classification (*n* = 162), resectable tumor status (*n* = 81), durvalumab not received by patient (*n* = 56), durvalumab therapy ongoing at end of study (*n* = 43), and a documented racial identity other than White or Black (*n* = 11) (exclusion criteria were not mutually exclusive). A total of 924 patients (White: *n* = 726, Black: *n* = 198) were analyzed in this study (Supplementary Fig. S2).

### Patient and Prior CRT Characteristics

Patient baseline characteristics are listed in Table S1. Black patients were younger than White patients (median age 67 years [IQR, 63-71] vs. 70 years [IQR, 65-73]; *P* < .01). Both groups were predominately male (White: 96%, Black: 94%; *P* = .29) and had similar median age-adjusted Charlson scores (White: 6 [5-7], Black: 6 [5-7]; *P* = .3), but Black patients were more likely to have chronic liver disease (22% vs. 9%; *P* < .01) and dementia (4% vs. 1%; *P* = .01) and less likely to have chronic obstructive pulmonary disease (63% vs. 72%; *P* = .01). Black patients were more likely to be current smokers (53% vs. 44%; *P* = .03), but other baseline characteristics including ECOG scores, NSCLC histological subtype, and PD-L1 tumor expression level were similar between the groups. There were no statistically significant differences in VA priority groups between the groups, but Black patients were more likely to live less than 50 miles from their cancer care than White patients (90% vs. 81%; *P* < .01).

Prior CRT and durvalumab initiation and delays, by race, are listed in Supplementary Table S2. Overall, there were no significant differences in CRT therapy between the groups. The majority of patients received carboplatin-based chemotherapy (White: 87%, Black: 89%; *P* = .35), with carboplatin/paclitaxel the most common regimen (White: 77%, Black: 76%; *P* = .75). The median number of radiation fractions between the groups was similar (White: 30 [30-33], Black: 30 [30-32]; *P* = .72) and most patients received a radiation dose between 54-66 Gy (White: 78%, Black: 79%; *P* = .54). The median number of days to imaging following CRT was similar between the groups (White: 30 days [20-43], Black: 30 days [21-44]; *P* = .45) and most patients (White: 66%, Black: 70%; *P* = .22) had a partial response to CRT (Supplementary Table S2).

### Durvalumab Treatment Patterns

**Figure 1.**
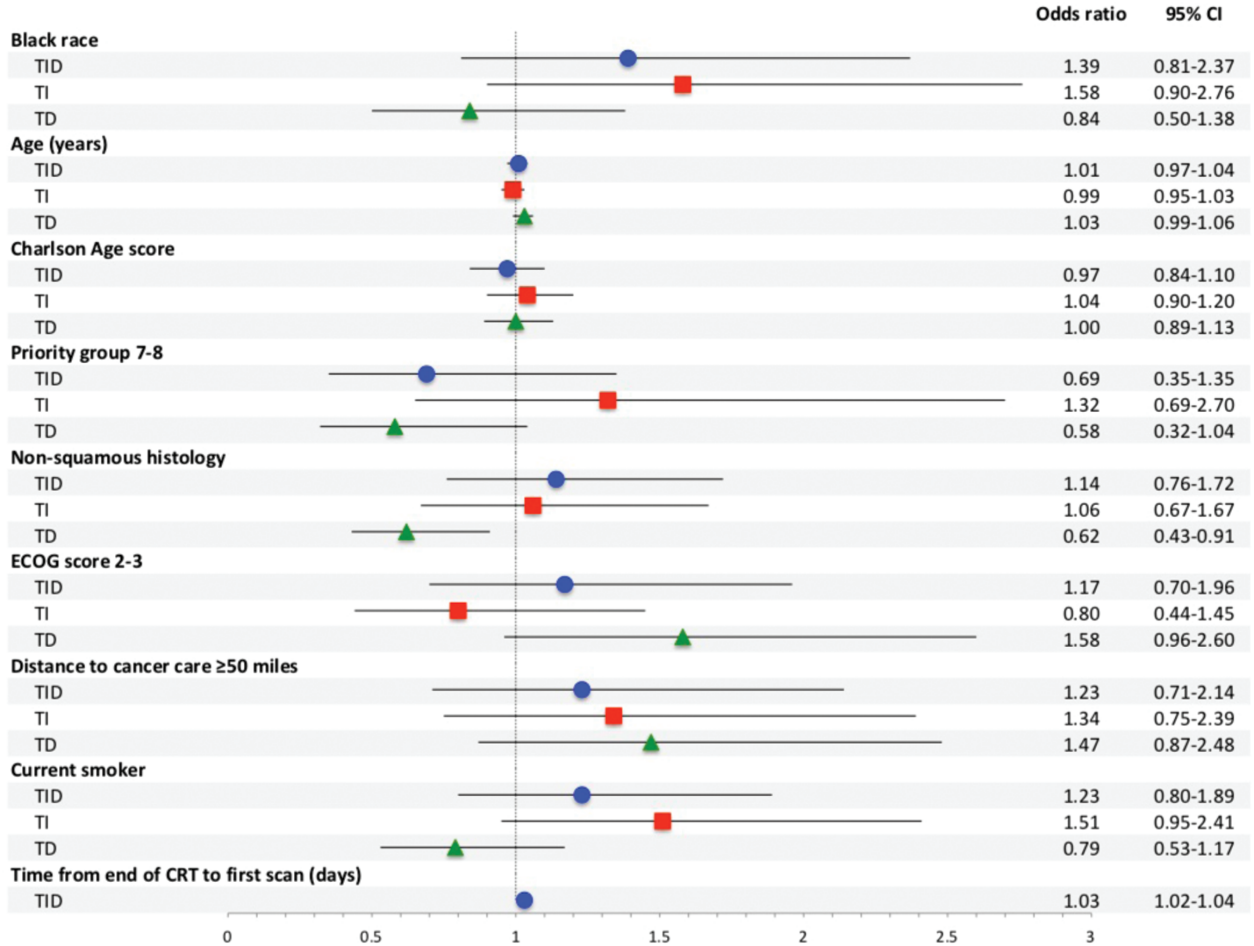
Multivariable logistic regression analysis of risk factors for TID, TI, and TD. TID, treatment initiation delay; TI, treatment interruption; TD, treatment discontinuation; CI, confidence interval.

There was no statistical difference between groups in proportion of patients that experienced a durvalumab TID, although Black patients experienced a TID numerically more often than White patients (Black: 45%, White: 38%; *P* = .07) (Table 1). In a multivariable logistic regression model with TID as the dependent variable, race was not an independent predictor of TID (OR, 1.39; 95% CI, 0.81-2.37). However, the time to first scan following the end of CRT was an independent risk factor for experiencing a TID, with odds of a TID increasing by 1.03 for each additional day (OR, 1.03; 95% CI, 1.02-1.04) (Fig. 1). Of patients that experienced TID, the median number of days of treatment delay was similar between groups (White: 61 [49-80], Black: 60 [51-84]; *P* = .48) (Supplementary Table S2). Physician preference was the most common documented reason associated with TID (White: 4.4%, Black: 4.0%, *P* = .49), though there were no significant differences in associated reasons for TID between the groups (Table 2).

**Table 1.** Patient treatment patterns, by race.

Characteristic	White (*n* = 726)	Black (*n* = 198)	*P*-value
Patients with durvalumab treatment initiation delay^a^, *n* (%)	275 (38)	89 (45)	.07
Patients with durvalumab treatment interruptions^b^, *n* (%)	130 (18)	49 (25)	***.03***
Durvalumab treatment discontinuations, *n* (%)	437 (60)	106 (54)	.09

^a^Durvalumab treatment delay was defined as more than 42 days from end of CRT to initiation of durvalumab.

^b^Durvalumab treatment interruptions were defined as more than 28 days between durvalumab infusions.

Bold italic value indicates statistical significance.

**Table 2.** Reported reasons associated with patient treatment outcomes, by race.

Characteristic	TID		TI^a^		TD	
	White	Black	White	Black	White	Black
Patient preference	2.1%	3.0%	1.0%	2.5%	5.6%	6.6%
Physician preference	4.4%	4.0%	1.0%	2.0%	1.2%	1.0%
Decline in performance status	2.5%	1.0%	1.1%	0.5%	1.9%	2.0%
Toxicity	2.3%	2.0%	7.7%	8.6%	20.0%	12.1%
Progression	0.1%	0.0%	0.6%	1.0%	24.4%	24.2%
Death	—	—	—	—	4.1%	3.0%
System issues	1.9%	2.5%	0.3%	1.0%	—	—
Social reasons	1.4%	3.5%	2.2%	4.0%	0.4%	0.5%
Insurance related	—	—	0.3%	0.0%	—	—
Other	6.5%	6.1%	5.5%	6.1%	2.1%	3.0%

^a^One reason was reported per treatment interruption; patients could have more than one treatment interruption during the course of therapy.

Abbreviations: TID, treatment initiation delay; TI, treatment interruption; TD, treatment discontinuation.

Overall, there were no significant differences between White and Black patients in the median number of doses received (White: 15 [7-24], Black: 18 [7-25]; *P* = .25). Black patients had a numerically longer DOT than White patients (White: 8.7 months [2.9-11.8], Black: 9.8 months [3.6-12.0]; *P* = .08). Black patients were more likely to experience TI than White patients (25% vs. 18%; *P* = .03) (Table 1); however, when covariates were included in a multivariable logistic regression model, Black race was not independently associated with TI (OR, 1.58; 95% CI, 0.90-2.76) and no other covariates were predictive of TI (Fig. 1). The median duration of a TI was similar between groups (White: 50 days [40-84], Black: 56 days [38-95]; *P* = .85). After adjusting for the number of days contributed by TIs (corrected DOT), the difference in DOT narrowed between the groups (White: 8.1 months [2.8-11.7], Black: 9.0 months [3.1-11.7]; *P* = .21), although Black patients still had numerically longer corrected DOT (Supplementary Table S3). Toxicity was the most commonly reported reason for TI in both groups (White: 8%, Black: 9%; *P* = .31), but there were no significant differences in reasons associated with TI between the groups (Table 2).

Sixty percent of White patients and 54% of Black patients discontinued durvalumab treatment (*P* = .09) (Table 1). Black race was not independently predictive of TD in a multivariable logistic regression model (OR, 0.84; 95% CI, 0.50-1.38), but non-squamous histology was associated with a lower risk of TD (OR, 0.62; 95% CI, 0.43-0.91) (Figure 1). Progression was the most commonly associated reason for TD in both groups (White: 24%, Black: 24%; *P* = .37). Toxicity was also commonly associated with TD, although White patients were more likely to discontinue due to toxicity compared to Black patients (20% vs. 12%; *P* = .04) There were no other significant differences in reasons associated with TD between groups (Table 2).

### Adverse Events

Sixty-one percent of White patients and 55% of Black patients experienced an adverse event while on durvalumab therapy (*P* = .12). Black patients were less likely to experience an immune-related adverse event than White patients (28% vs. 36%; *P* = .03) and, specifically, were less likely to experience pneumonitis (7% vs. 14%; *P* < .01). Black patients were also less likely to experience non-immune-related adverse events when compared to White patients (34% vs. 42%; *P* = .04) (Supplementary Table S4).

## Discussion

Clinical trials should represent the diversity of the population the therapeutic is intended to treat, but despite having a disproportionate share of the burden of many diseases, including cancer, racial and ethnic minorities are historically underrepresented in clinical trials.^24-26^ In immunotherapy trials the inclusion of minority populations is particularly relevant as data are emerging that there might be differences between racial groups in the way the immune system combats cancer.^27^ These differences can be attributed to a variety of etiologies, notably that societal and systemic stressors are linked to chronic inflammation, which can drive immune dysregulation and poor health outcomes among patients from disadvantaged communities.^28,29^

Given that racial minorities and patients with limited access to healthcare systems are underrepresented in clinical trials,^22^ it is imperative for these patients to be included in real-world studies to gain a complete picture of medication use in practice. Historically, in real-world practice, Black patients have been less likely to receive immunotherapy,^30,31^ so have patients who are uninsured, on Medicaid, or in area-level poverty.^30-36^ However, of those who do receive immunotherapy, real-world studies have shown that outcomes are the same as or better than White patients. In late-stage NSCLC, receipt of pembrolizumab, an anti-programmed cell death protein 1 (PD-1) monoclonal antibody, was associated with similar progression-free survival and overall survival between Black and White patients.^21^ Another study of patients with NSCLC receiving anti-PD-L1/anti-PD-1 monoclonal antibodies found that Black patients had improved outcomes when compared to White patients, with longer time-to-discontinuation and longer overall survival.^22^ A pattern of improved survival in Black patients upon receipt of immunotherapy has also been seen in other types of cancers.^31,34^ This improved efficacy of immunotherapy as seen in these studies presents an interesting conundrum, as it is in contrast with other literature suggesting a negative relationship between immunotherapy efficacy and higher inflammatory burden as seen in disadvantaged populations. Many hypotheses have been generated regarding this apparent inconsistency, including that Black patients might have a lower incidence of hyperprogressive disease with anti-PD-1/PD-L1 therapy,^22^ and/or may derive benefit from a higher tumor mutational burden and genomic instability as correlated with smoking status that appears to favor ICI efficacy.^37-41^

Black patients and patients of other racial and ethnic minorities generally had worse overall comorbidities when compared to White patients^42^; such was the case in this study too. Black patients in this study were also more likely to live closer to a site of cancer care, indicating a lower travel burden which has been associated with improved treatment^43,44^; however, access to transportation, which impacts access-to-care, was not evaluated in this study. Black and White patients in this study had similar prior CRT characteristics and these were not predictive of TID. Time from the end of CRT to the first CT or PET scan was predictive of TID, suggesting that access to timely post-CRT imaging could be a barrier to initiating durvalumab therapy. This might be improved for all patients by increasing access to patient navigators to improve coordination of cancer services, although the clinical implications of TID remain undetermined.^45-47^

Race was also not predictive of TI or TD in multivariable logistic regression models. Toxicity was the major contributor to TI in both patient groups. Toxicity and progression were also major risk factors for TD in both groups. Non-squamous histology was associated with a lower risk of TD, which corroborates the finding that squamous histology has been shown to be associated with worse outcomes.^48^ Smoking status has also been shown to be associated with a higher risk of immune-related adverse events,^49^ but in this study, Black patients were less likely to have reported immune-related adverse events and particularly were less likely to have pneumonitis. This pattern has been duplicated in other studies,^22,50^ and it has been postulated that Black patients have longer treatment durations partially due to reduced toxicity to ICI therapy.^22^ In this study, Black patients were less likely to discontinue treatment due to toxicity, further supporting this hypothesis. However, due to the retrospective nature of this study, the possibility exists that there may be documentation bias in adverse event reporting, which may falsely lower the reported proportion of patients who experienced these events.

The predominant underlying factor in healthcare disparities is access to quality care, in which appears to be mitigated in systems providing equal access-to-care.^15,51,52^ The VHA is the largest integrated healthcare system for cancer care in the US acting as an equal-access system, which is known to reduce disparities in care when compared to other health systems in the US.^53-55^ The results of this study corroborate these prior findings and suggest that disparities in durvalumab treatment patterns are mitigated in the VHA. However, although the VHA eliminates many access-related barriers to care, particularly by mitigating costs associated with services, it cannot completely eliminate residual disparities outside of the healthcare system, including access to housing, food, transportation, and other essential services. There still remain major gaps in understanding the ways in which health outcomes are shaped by structural, socioeconomic, and socio-environmental factors in historically disadvantaged communities.^56,57^

There are limitations in this study. Primarily, race is a social construct and observable differences in outcomes between races are possibly due to the consequences of social stratification in the US and the historical inequitable distribution of resources that cause socioeconomic disparities in healthcare delivery.^21^ A more robust depiction of health disparities would take an intersectional approach and include additional examinations of social determinants of health, including healthcare quality, education, and economic stability, although we did mitigate some of these factors by inclusion of travel burden assessments and VA priority group as a measure of SES. Given that newer immunotherapies can be financially taxing to both patients and payers,^58^ it would be expected that there would be inequities in immunotherapy treatment availability to disadvantaged populations. The VHA, as an equal-access health system, limits the generalizability of the financial burden of immunotherapy to the general US population. Additionally, due to the makeup of the veteran population, which is majority-White and majority-male, as well as limitations in race and ethnicity reporting within the VHA EHR, we were only able to evaluate racial disparities between Black and White patients. Furthermore, PD-L1 tumor expression level was unknown for more than 80% of the patients in the study. Durvalumab effectiveness might be influenced by PD-L1 tumor expression level so this warrants exploration in future studies. Fortunately, PD-L1 tumor expression level was well-balanced between groups for the patients for whom it was known in this study. Also, we did not exclude patients with other concurrent cancers, receipt of other chemotherapies or immunotherapies prior to durvalumab, or those who had progressed after concurrent chemoradiotherapy. Finally, this study was only able to assess treatment patterns in patients that were prescribed and received durvalumab. Previous literature has shown that Black patients have reduced rates of receiving guideline-concordant care^17,59,60^; the design of this study was unable to assess equal receipt of appropriate treatment and we are unable to comment if there is a racial disparity in prescribing patterns or patient management, including safety assessments/interventions.

## Conclusions

Race was not found to be linked with durvalumab treatment patterns, including treatment initiation delay, interruption, and discontinuation in this real-world study of patients with unresectable stage III NSCLC treated at the VHA.

## Supplementary Material

Supplementary material is available at *The Oncologist* online.

oyad172_suppl_Supplementary_Materials

## Data Availability

The data underlying this article will be shared on reasonable request to the corresponding author.
