# Supplementary material for: Health Equity in Patients Receiving Durvalumab for Unresectable Stage III Non-Small Cell Lung Cancer in the US Veterans Health Administration
Source: Oncologist. 2023 Jun 19;28(9):804–11. doi: 10.1093/oncolo/oyad172 (PMC10485300; doi:10.1093/oncolo/oyad172)
Supplement: oyad172_suppl_Supplementary_Materials [file oyad172_suppl_supplementary_materials.zip › Supp_Figure2.pdf]

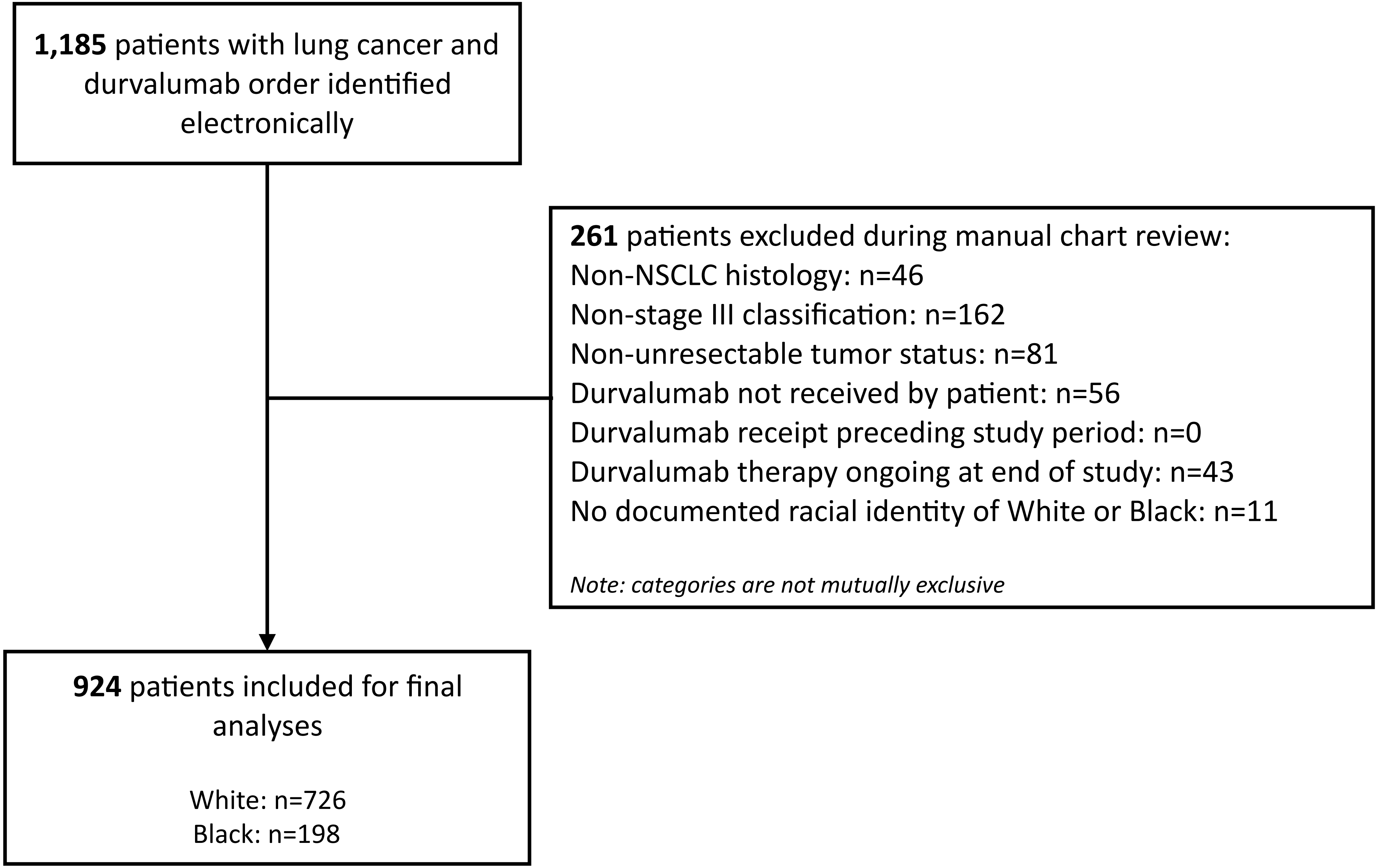
1,185 patients with lung cancer and durvalumab order identified electronically
261 patients excluded during manual chart review:
Non-NSCLC histology: n=46
Non-stage III classification: n=162
Non-unresectable tumor status: n=81
Durvalumab not received by patient: n=56
Durvalumab receipt preceding study period: n=0
Durvalumab therapy ongoing at end of study: n=43
No documented racial identity of White or Black: n=11
Note: categories are not mutually exclusive
924 patients included for final analyses
White: n=726
Black: n=198
