# Supplementary material for: Health Equity in Patients Receiving Durvalumab for Unresectable Stage III Non-Small Cell Lung Cancer in the US Veterans Health Administration
Source: Oncologist. 2023 Jun 19;28(9):804–11. doi: 10.1093/oncolo/oyad172 (PMC10485300; doi:10.1093/oncolo/oyad172)
Supplement: oyad172_suppl_Supplementary_Materials [file oyad172_suppl_supplementary_materials.zip › Supp_Table1_ccfp.docx]

**Supplemental Table 1.** Patient baseline characteristics, by race

| **Characteristic** | **White**  **(n=726)** | **Black**  **(n=198)** | **P-value** |
| --- | --- | --- | --- |
| Age (years), median (IQR) | 70 (65-73) | 67 (63-71) | ***<0.0001*** |
| Age groups, n (%) | -- | -- | -- |
| Missing/unknown | 0 (0) | 0 (0) | -- |
| <65 years | 158 (22) | 69 (35) | ***0.0001*** |
| 65-74 years | 451 (62) | 107 (54) | ***0.0393*** |
| >74 years | 117 (16) | 22 (11) | 0.0923 |
| Male, n (%) | 695 (96) | 186 (94) | 0.2890 |
| Charlson score, median (IQR) | 3 (3-5) | 4 (3-5) | 0.5253 |
| Age-adjusted Charlson score, median (IQR) | 6 (5-7) | 6 (5-7) | 0.2955 |
| Selected comorbidities, n (%) | -- | -- | -- |
| Congestive heart failure | 97 (13) | 24 (12) | 0.6467 |
| COPD | 525 (72) | 125 (63) | ***0.0122*** |
| Cerebrovascular disease | 110 (15) | 21 (11) | 0.1041 |
| Dementia | 8 (1) | 7 (4) | ***0.0250*** |
| Diabetes | 230 (32) | 71 (36) | 0.2662 |
| Hemi/paraplegia | 6 (1)) | 3 (2) | 0.4126 |
| HIV/AIDS | 3 (<1) | 2 (1) | 0.2920 |
| Liver | 63 (9) | 44 (22) | ***<0.0001*** |
| Myocardial infarction | 47 (6) | 16 (8) | 0.4265 |
| Peptic ulcer disease | 12 (2) | 4 (2) | 0.7586 |
| Peripheral vascular disease | 176 (24) | 39 (20) | 0.1797 |
| Renal disease | 77 (11) | 29 (15) | 0.1138 |
| VA priority group, % | -- | -- | -- |
| Missing/unknown | 194 (27) | 61 (31) | -- |
| Group 1 | 189 (26) | 42 (21) | 0.2851 |
| Groups 2-6 | 281 (39) | 84 (42) | 0.0750 |
| Groups 7-8 | 62 (8) | 11 (6) | 0.2249 |
| Distance from site of cancer care (miles), median (IQR) | 19 (9-41) | 10 (5-23) | ***<0.0001*** |
| Distance from site of cancer care (miles), n (%) | -- | -- | -- |
| Missing/unknown | 1 (<1) | 0 (0) | -- |
| <50 miles | 589 (81) | 178 (90) | ***0.0040*** |
| ≥50 miles | 136 (19) | 20 (10) |  |
| Smoking status, n (%) | -- | -- | -- |
| Missing/unknown | 20 (3) | 4 (2) | -- |
| Current | 317 (44) | 104 (53) | ***0.0313*** |
| Former | 380 (52) | 90 (45) | 0.0664 |
| Never | 9 (1) | 0 (0) | 0.2178 |
| ECOG performance status, n (%) | -- | -- | -- |
| Missing/unknown | 132 (18) | 49 (25) | -- |
| 0-1 | 471 (65) | 121 (61) | 0.6035 |
| 2-3 | 123 (17) | 28 (14) |  |
| NSCLC subtype, n (%) | -- | -- | -- |
| Missing/unknown | 37 (5) | 18 (9) | -- |
| Squamous cell | 377 (52) | 87 (44) | 0.1263 |
| Non-squamous cell | 303 (42) | 92 (46) | 0.0870 |
| Mixed | 9 (1) | 1 (1) | 0.6968 |
| PD-L1 tumor expression level, n (%) | -- | -- | -- |
| Missing/unknown | 579 (80) | 165 (83) | -- |
| PD-L1 expression level <1% | 46 (6) | 13 (7) | 0.3703 |
| PD-L1 expression level ≥1% | 101 (14) | 20 (10) |  |

IQR=interquartile range, COPD=chronic obstructive pulmonary disease, HIV=human immunodeficiency virus, AIDS=acquired immunodeficiency syndrome, VA=Veterans Affairs, ECOG=Eastern Cooperative Oncology Group, NSCLC=non-small cell lung cancer, PD-L1=programmed death-ligand 1
