# Supplementary material for: Health Equity in Patients Receiving Durvalumab for Unresectable Stage III Non-Small Cell Lung Cancer in the US Veterans Health Administration
Source: Oncologist. 2023 Jun 19;28(9):804–11. doi: 10.1093/oncolo/oyad172 (PMC10485300; doi:10.1093/oncolo/oyad172)
Supplement: oyad172_suppl_Supplementary_Materials [file oyad172_suppl_supplementary_materials.zip › Supp_Table2_ccfp.docx]

**Supplemental Table 2.** Prior chemoradiation therapy and durvalumab initiation and delays, by race

| **Characteristic** | **White**  **(n=726)** | **Black**  **(n=198)** | **P-value** |
| --- | --- | --- | --- |
| Prior chemotherapy, n (%) | -- | -- | -- |
| Missing/unknown | 0 (0) | 0 (0) | -- |
| Cisplatin-based chemotherapy | 84 (12) | 20 (10) | 0.5620 |
| Carboplatin-based chemotherapy | 631 (87) | 177 (89) | 0.3506 |
| Other | 11 (1) | 1 (1) | 0.4783 |
| Total chemotherapy weeks, median (IQR) | 6 (5-7) | 6 (4-6) | 0.0821 |
| Radiation dose (Gy), n (%) | -- | -- | -- |
| Missing/unknown | 77 (11) | 22 (11) | -- |
| <54 | 21 (3) | 5 (3) | 1.0000 |
| 54-66 | 564 (78) | 156 (79) | 0.5405 |
| 67-74 | 59 (8) | 13 (6) | 0.4773 |
| >74 | 5 (<1) | 2 (1) | 0.6453 |
| Radiation fractions, median (IQR) | 30 (30-33) | 30 (30-32) | 0.7173 |
| Chemoradiation therapy type, n (%) | -- | -- | -- |
| Missing/unknown | 0 (0) | 0 (0) | -- |
| Sequential | 12 (2) | 3 (1) | 1.0000 |
| Concurrent | 714 (98) | 195 (99) |  |
| Time from end of CRT to first scan (days), median (IQR) | 30 (20-43) | 30 (21-44) | 0.4543 |
| Time from end of CRT to first scan, n (%) | -- | -- | -- |
| Missing/unknown | 105 (14) | 21 (11) | -- |
| <2 weeks | 84 (12) | 19 (10) | 0.3284 |
| 2 weeks to <4 weeks | 172 (24) | 52 (26) | 0.6606 |
| 4 weeks to <6 weeks | 204 (28) | 53 (27) | 0.4653 |
| 6 weeks to <8 weeks | 69 (9) | 28 (14) | 0.0908 |
| 8 weeks to <10 weeks | 35 (5) | 9 (4) | 0.7768 |
| ≥10 weeks | 57 (8) | 16 (8) | 0.9548 |
| Chemoradiation therapy response^a^, n (%) | -- | -- | -- |
| Missing/unknown | 110 (15) | 31 (16) | -- |
| Complete response | 25 (4) | 6 (3) | 0.7843 |
| Partial response | 479 (66) | 138 (70) | 0.2192 |
| Stable disease | 81 (11) | 15 (8) | 0.1716 |
| Progressive disease | 22 (3) | 5 (2) | 1.0000 |
| Non-evaluable | 9 (1) | 3 (1) | 0.7259 |
| Time to durvalumab initiation (days), median (IQR) | 38 (28-53) | 41 (30-57) | 0.0511 |
| Patients with durvalumab treatment initiation delay^b^, n (%) | 275 (38) | 89 (45) | 0.0711 |
| Days of treatment initiation delay^c^, median (IQR) | 61 (49-80) | 60 (51-84) | 0.4783 |

IQR=interquartile range

^a^CRT response for patients with scan documented between 2 weeks before CRT end and up to 2 weeks after durvalumab initiation

^b^Durvalumab treatment delay defined as more than 42 days from end of CRT to initiation of durvalumab

^c^Days of treatment initiation delay only calculated for patients with a documented TID
