# Supplementary material for: Health Equity in Patients Receiving Durvalumab for Unresectable Stage III Non-Small Cell Lung Cancer in the US Veterans Health Administration
Source: Oncologist. 2023 Jun 19;28(9):804–11. doi: 10.1093/oncolo/oyad172 (PMC10485300; doi:10.1093/oncolo/oyad172)
Supplement: oyad172_suppl_Supplementary_Materials [file oyad172_suppl_supplementary_materials.zip › Supp_Table3_ccfp.docx]

**Supplemental Table 3.** Durvalumab therapy, by race

| **Characteristic** | **White**  **(n=726)** | **Black**  **(n=198)** | **P-value** |
| --- | --- | --- | --- |
| Durvalumab start of therapy by year, n (%) | -- | -- | -- |
| 2017 | 11 (1) | 1 (1) | 0.4783 |
| 2018 | 251 (35) | 58 (29) | 0.1627 |
| 2019 | 347 (48) | 96 (48) | 0.8635 |
| 2020^a^ | 117 (16) | 43 (22) | 0.0648 |
| Durvalumab duration of therapy (DOT) (months), median (IQR) | 8.7 (2.9-11.8) | 9.8 (3.6-12.0) | 0.0829 |
| Durvalumab DOT (months), n (%) | -- | -- | -- |
| Missing/unknown | 6 (1) | 2 (1) | -- |
| 0-3 months | 183 (25) | 44 (22) | 0.3936 |
| 3-6 months | 95 (13) | 23 (11) | 0.5886 |
| 6-9 months | 91 (13) | 21 (11) | 0.4659 |
| 9-12 months | 227 (31) | 65 (33) | 0.6631 |
| >12 months | 124 (17) | 43 (22) | 0.1295 |
| Durvalumab total doses/infusions, median (IQR) | 15 (7-24) | 18 (7-25) | 0.2458 |
| Patients with durvalumab treatment interruptions^b^, n (%) | 130 (18) | 49 (25) | ***0.0308*** |
| Number of durvalumab treatment interruptions, median (IQR) | 1 (1-1) | 1 (1-1) | 0.7446 |
| Days contributed by durvalumab treatment interruptions, median (IQR) | 50 (40-84) | 56 (38-95) | 0.8479 |
| Durvalumab corrected DOT (months)^c^, median (IQR) | 8.1 (2.8-11.7) | 9.0 (3.1-11.7) | 0.2117 |
| Durvalumab treatment discontinuations, n (%) | 437 (60) | 106 (54) | 0.0916 |
| Completed planned treatment, n (%) | 289 (40) | 92 (46) |  |

IQR=interquartile range

^a^Enrollment ended on 06/30/2020

^b^Durvalumab treatment interruptions defined as more than 28 days between durvalumab infusions

^c^Durvalumab corrected duration of therapy defined as DOT minus days contributed by treatment interruptions
