# Supplementary material for: Health Equity in Patients Receiving Durvalumab for Unresectable Stage III Non-Small Cell Lung Cancer in the US Veterans Health Administration
Source: Oncologist. 2023 Jun 19;28(9):804–11. doi: 10.1093/oncolo/oyad172 (PMC10485300; doi:10.1093/oncolo/oyad172)
Supplement: oyad172_suppl_Supplementary_Materials [file oyad172_suppl_supplementary_materials.zip › Supp_Table4_ccfp.docx]

**Supplemental Table 4.** Selected adverse events, by race

| **Characteristic** | **White**  **(n=726)** | **Black**  **(n=198)** | **P-value** |
| --- | --- | --- | --- |
| Immune- and non-immune-related adverse events, n (%) | 440 (61) | 108 (55) | 0.1239 |
| Immune-related adverse events, n (%) | 260 (36) | 551 (28) | ***0.0345*** |
| Pneumonitis | 103 (14) | 13 (7) | ***0.0041*** |
| Hepatitis | 7 (1) | 3 (2) | 0.4540 |
| Colitis | 14 (2) | 4 (2) | 1.0000 |
| Endocrinopathies | 100 (14) | 20 (10) | 0.1729 |
| Nephritis | 6 (1) | 3 (2) | 0.4126 |
| Dermatologic | 44 (6) | 10 (5) | 0.5912 |
| Other immune-related condition | 30 (4) | 7 (4) | 0.7042 |
| Non-immune-related adverse events, n (%) | 305 (42) | 67 (34) | ***0.0377*** |
| Cough | 42 (6) | 8 (4) | 0.3361 |
| Fatigue | 146 (20) | 27 (14) | 0.0385 |
| Radiation pneumonitis | 32 (4) | 7 (4) | 0.5884 |
| Infection | 13 (2) | 1 (1) | 0.3234 |
| Dyspnea | 64 (9) | 13 (7) | 0.3100 |
| Rash | 30 (4) | 15 (8) | 0.0460 |
| Diarrhea | 29 (4) | 6 (3) | 0.5287 |
| Abdominal pain | 3 (<1) | 1 (1) | 1.0000 |
| Muscle pain | 15 (2) | 1 (1) | 0.2165 |
| Other non-immune-related condition | 108 (15) | 25 (13) | 0.4241 |
