# Supplementary figures and images for: Health Equity in Patients Receiving Durvalumab for Unresectable Stage III Non-Small Cell Lung Cancer in the US Veterans Health Administration
Source: Oncologist. 2023 Jun 19;28(9):804–11. doi: 10.1093/oncolo/oyad172 (PMC10485300; doi:10.1093/oncolo/oyad172)

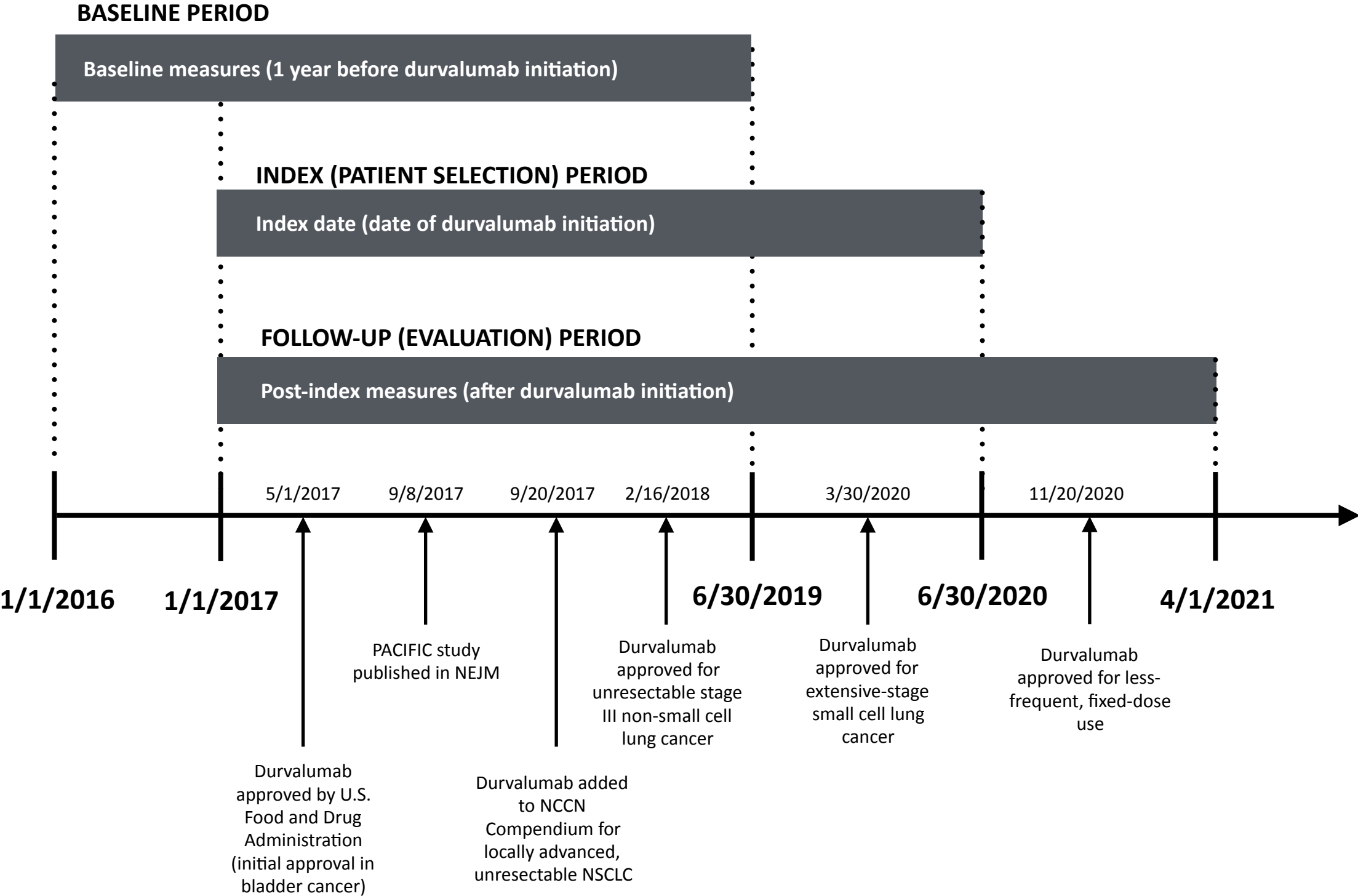

Supplement: oyad172_suppl_Supplementary_Materials [file oyad172_suppl_supplementary_materials.zip › Supp_Figure1.pdf]
